# A nomogram for predicting survival of nasopharyngeal carcinoma patients with metachronous metastasis

**DOI:** 10.1097/MD.0000000000004026

**Published:** 2016-07-08

**Authors:** Zixun Zeng, Lujun Shen, Yue Wang, Feng Shi, Chen Chen, Ming Wu, Yutong Bai, Changchuan Pan, Yunfei Xia, Peihong Wu, Wang Li

**Affiliations:** a Zhong Shan Medical School, Sun Yat-sen University; b Department of Medical Imaging and Interventional Radiology, Sun Yat-sen University Cancer Center; c Collaborative Innovation Center of Cancer Medicine, Sun Yat-sen University; d Department of Radiation Oncology, Sun Yat-sen University Cancer Center, Guangzhou; e Department of Medical Oncology, Sichuan Cancer Hospital and Institute, Chengdu, People's Republic of China.

**Keywords:** metastasis, nasopharyngeal carcinoma, nomogram, prognosis

## Abstract

Patients with metachronous metastatic nasopharyngeal carcinoma (NPC) differ significantly in survival outcomes. The aim of this study is to build a clinically practical nomogram incorporating known tumor prognostic factors to predict survival for metastatic NPC patients in epidemic areas.

A total of 860 patients with metachronous metastatic nasopharyngeal carcinoma were analyzed retrospectively. Variables assessed were age, gender, body mass index, Karnofsky Performance Status (KPS), Union for International Cancer Control (UICC) T and N stages, World Health Organization (WHO) histology type, serum lactate dehydrogenase (sLDH) level, serum Epstein–Barr virus (EBV) level, treatment modality, specific metastatic location (lung/liver/bone), number of metastatic location(s) (isolated vs multiple), and number of metastatic lesion(s) in metastatic location(s) (single vs multiple). The independent prognostic factors for overall survival (OS) by Cox-regression model were utilized to build the nomogram.

Independent prognostic factors for OS of metastatic NPC patients included age, UICC N stage, KPS, sLDH, number of metastatic locations, number of metastatic lesions, involvement of liver metastasis, and involvement of bone metastasis. Calibration of the final model suggested a c-index of 0.68 (95% confidence interval [CI], 0.65–0.69). Based on the total point (TP) by nomogram, we further subdivided the study cohort into 4 groups. Group 1 (TP < 320, 208 patients) had the lowest risk of dying. Discrimination was visualized by the differences in survival between these 4 groups (group 2/group 1: hazard ratio [HR] = 1.61, 95%CI: 1.24–2.09; group 3/group 1: HR = 2.20, 95%CI: 1.69–2.86; and group 4/group 1: HR = 3.66, 95%CI: 2.82–4.75).

The developed nomogram can help guide the prognostication of patients with metachronous metastatic NPC in epidemic areas.

## 1 Introduction

Nasopharyngeal carcinoma (NPC) accounts for 80,000 new cases and 50,000 deaths annually, ranking 11th among all malignancies in China.^[1]^ Past reports showed that metastasis to distant sites account for 50% of treatment failure for NPC,^[2,3]^ while an increasing interest in the use of local therapy for metastases and a series of reports published showed that even long-term survival could be achieved for selective NPC patients with oligometastasis by combined systemic and local therapies.^[4–7]^ As cancer metastasis covers a wide spectrum of different conditions,^[8,9]^ it is attracting to explore the theoretical margin of the susceptible target patients worth aggressive management.

Patients presenting distant metastasis at the time of diagnosis of NPC are termed synchronous metastatic NPC patients, indicating stage IV. Other patients suffering distant metastasis over 6 months after radical therapy are termed metachronous metastatic NPC patients. To the best of our knowledge, there had been limited published reports on prognostic model of metachronous metastatic NPC patients. Due to the rarity of synchronous metastatic NPC patients, most published studies included both patients with metastases at diagnosis and subsequent after 6 months. However, compared with synchronous metastasis, patients with metachronous metastasis were 1.7 times more prevalent,^[10]^ underwent different treatment regimens, and had different survival rates, warranting a differentiated analysis.

Therefore, in this study, we aim to build a prognostic model for metachronous metastatic NPC patients predictive of overall survival (OS) after metastasis, and we further visualize it as nomogram for more friendly clinical practice.

## 2 Patients and method

### 2.1 Study population

We retrospectively reviewed 1648 NPC patients with distant metastasis treated at Sun Yat-sen University Cancer Center between January 1995 and December 2010. The inclusion criteria were as following: histologically confirmed primary NPC; distant metastases confirmed by histological evaluation, ultrasound, or chemotherapy (CT) of the abdomen during subsequent follow-up; and presence of complete pretreatment evaluation, including complete history, physical examination, hematology and biochemistry profiles, CT or magnetic resonance imaging scans of the head and neck regions, radiographs/CT scans of the chest, sonography/CT scans of the abdomen, and whole-body bone scan. The exclusion criteria were any of the following: presence of metastasis at diagnosis or presence of other malignancies, and refusal of treatment. The Hospital Ethics Committee in Sun Yat-sen University Cancer Center approved this study.

### 2.2 Treatment

All patients received multimodality treatment. A total of 439 (51.0%) patients received palliative chemotherapy as a systemic treatment after admission. The 1st-line regimen was nearly exclusively platinum-based, with cisplatin in combination with 1 or 2 of the following drugs: 5-fluorouracil, paclitaxel, gemcitabine, and bleomycin for 4 to 6 cycles. Treatment was discontinued by request of the patients or for intolerable drug toxicity; the median number of cycles was 4 (range 1–27). Other chemotherapy regimens included newer agents, such as gemcitabine and paclitaxel. Local therapies for distant metastasis consist of minimal invasive therapy, including trans-arterial chemoembolization, radiofrequency ablation, or both, radiotherapy, and surgery.

### 2.3 Study protocol

We retrospectively collected patients’ demographic, clinical, and therapeutic characteristics, collecting data of metastasis (number of lesions in metastatic locations, number of metastatic locations, liver/lung/bone involvement, etc.) We assessed the effect of age, gender,^[11]^ body mass index, and the Union of International Cancer Control (UICC) stage classification for T and N on metastatic survival. Metastatic OS was defined as the interval between the diagnosis of distant metastasis to the time of death or to the end of the study. Data from patients alive at the end of study (December 31st, 2010) were defined as censored. We verified survival status on August 31st, 2010 by direct telecommunication with the patient or family and checking the clinic attendance records.

### 2.4 Statistical methods

Statistical analyses were performed on IBM SPSS Statistics 20.0 (IBMCorp.) and R version 2.15.3 (R Foundation for Statistical Computing, Vienna, Austria. ISBN 3–900051–07–0, 187 URL http://www.R-project.org/)

Survival was estimated with the Kaplan–Meier method and was compared by using the log-rank test. Multiple regression analysis was undertaken by using Cox proportional hazard models. Alpha was set at 0.05, and all tests were 2 tailed. Backward stepdown selection process was utilized for the selection of covariates for the final model. To maximize the predictive ability of the model, all variables in the multivariable model were used to develop a prognostic nomogram using the linear predictor method (by the package of rms in R). The performance of nomogram was measured by concordance index (c-index) by using rcorrp.cens package in Hmisc in R and calibration through comparing nomogram-predicted survival with observed Kaplan–Meier estimated survival probability.^[12]^

## 3 Results

### 3.1 Patient characteristics

A total of 1648 patients were sort out as metastasis population. A total of 252 patients were excluded due to lack of appropriate full data, 536 patients were excluded due to synchronous metastasis. The remaining 860 patients met all criteria were enrolled for our study.

Patient characteristics are described in Table 1. The median age was 44 (ranging from 11 to 80 years), 689 male patients (80.1%) compared with 171 female patients (19.9%). Histologically, most patients were World Health Organization (WHO) III type NPC (803, 93.4%); the others were WHO II (38, 4.4%) and WHO I (12, 1.4%). The most frequent involved locations for metastases were bone (492, 57.2%), lung (433, 50.3%), and liver (346, 40.2%); isolated organ metastases were bone (446, 51.9%), lung (398, 46.3%), and liver (289, 33.6%). Multiple lesions (768, 89.3%) were detected more than single lesion (92, 10.6%). Single organ involvement accounted for over half (439, 51.0%) patients.

**Table 1:**
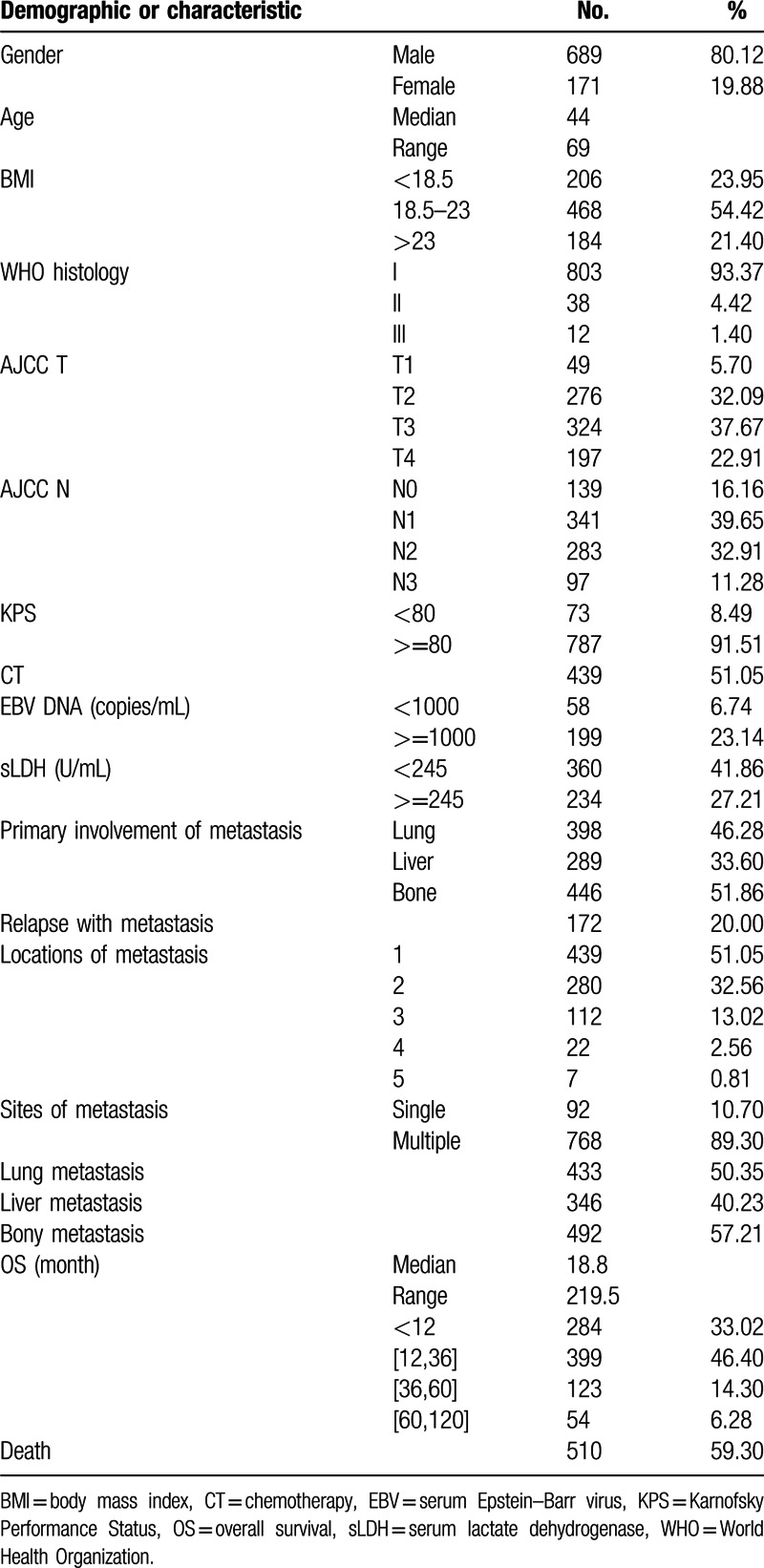
Basic characteristics: demographic clinicopathologic characteristics of patients with metachronous metastatic nasopharyngeal carcinoma in epidemic area.

### 3.2 Metastatic characteristics and survival

Overall, 510 patients (59.3%) died before last follow-up. The median follow-up time after the diagnosis of distant metastasis was 38 month. Median metastatic OS was 19 months (interquartile range [8–31] months), the 3- and 5-year OS rate of the study cohort were 20.7% and 6.3%, respectively.

On univariate analysis, age, body mass index, Karnofsky Performance Status (KPS), UICC N stage, serum lactate dehydrogenase (LDH) level, and metastasis characteristics (number of metastatic lesions, number of metastatic locations, liver metastasis, and bone metastasis) were identified as independent prognosticators for metastatic OS (Table 2).

**Table 2:**
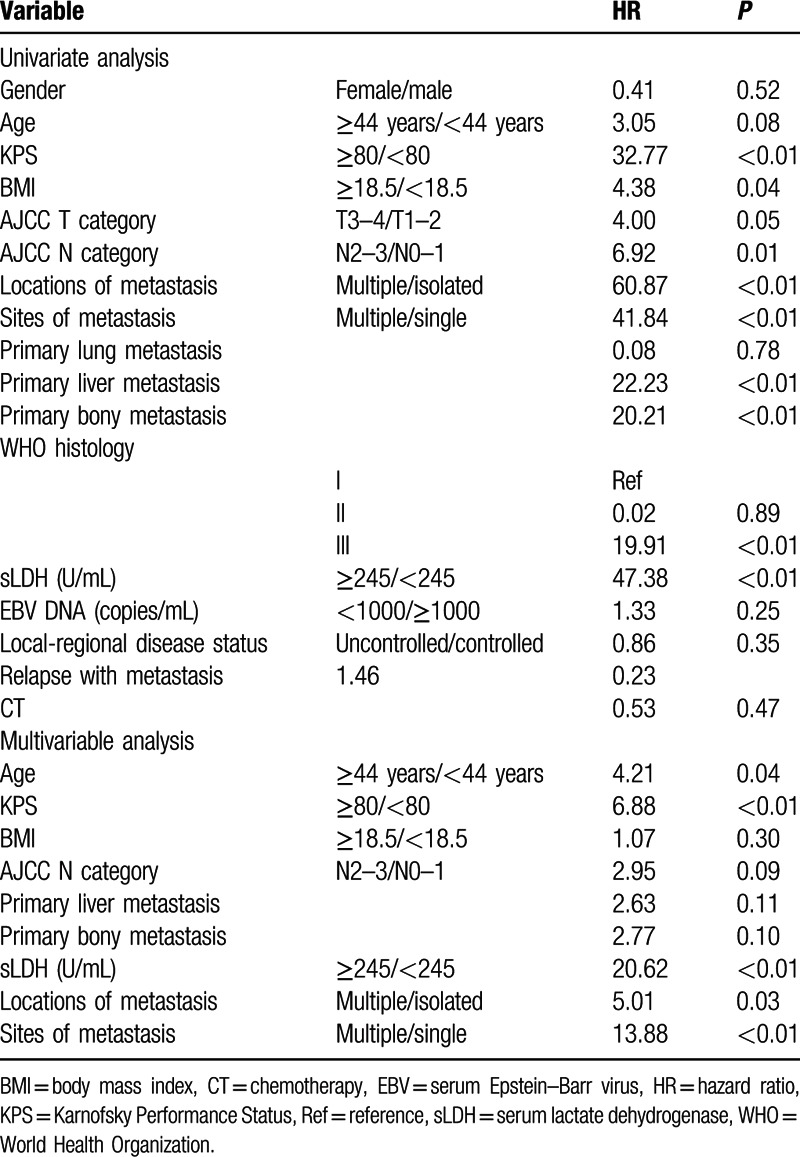
Univariable and multivariable analysis.

On multivariable analysis using backward method, age, KPS, UICC N stage, serum LDH level, and metastasis characteristics (number of metastatic lesions, number of metastatic sites, primary liver metastasis, and primary bone metastasis) remain significant (Table 2).

### 3.3 Prognostic nomogram for OS

**Figure 1:**
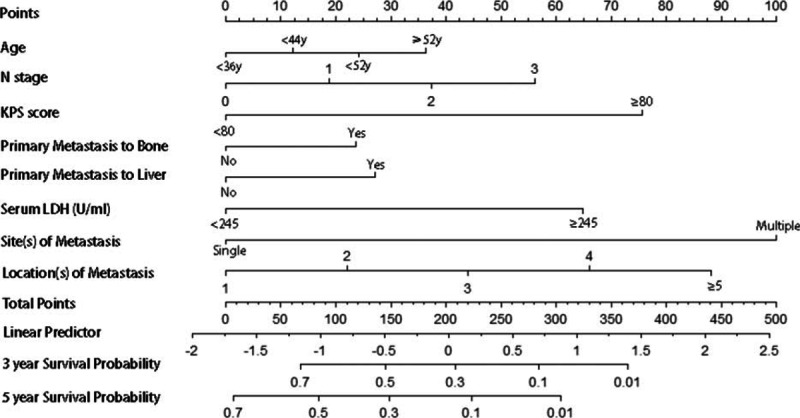
Metastatic OS nomogram for metachronous metastatic NPC patients. NCP = nasopharyngeal carcinoma, OS = overall survival.

**Figure 2:**
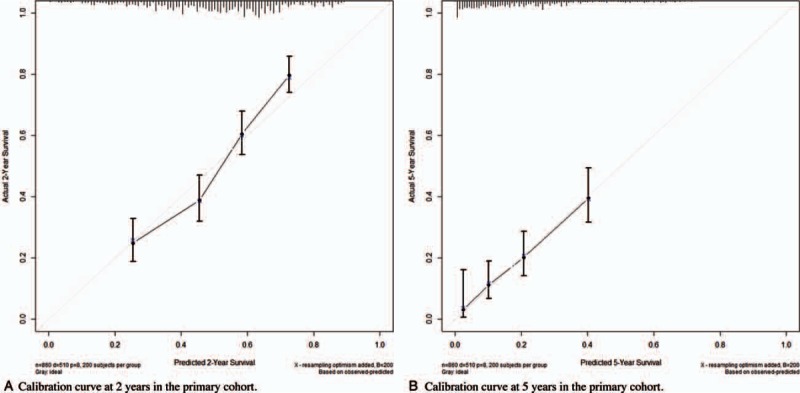
The calibration curves for predicting patient survival at 2, 3, and 5 years in the primary cohort.

We built a Cox regression model incorporating all independent prognostic factors for metastatic OS based on multivariable analysis, and further visualized it into a nomogram as shown in Fig. 1. The C-index for metastatic OS prediction was 0.68 (95% confidence interval [CI], 0.65–0.69). The calibration plot for the probability of survival at 3 or 5 year after surgery showed an optimal agreement between the prediction by nomogram and actual observation in Fig. 2.

**Figure 3:**
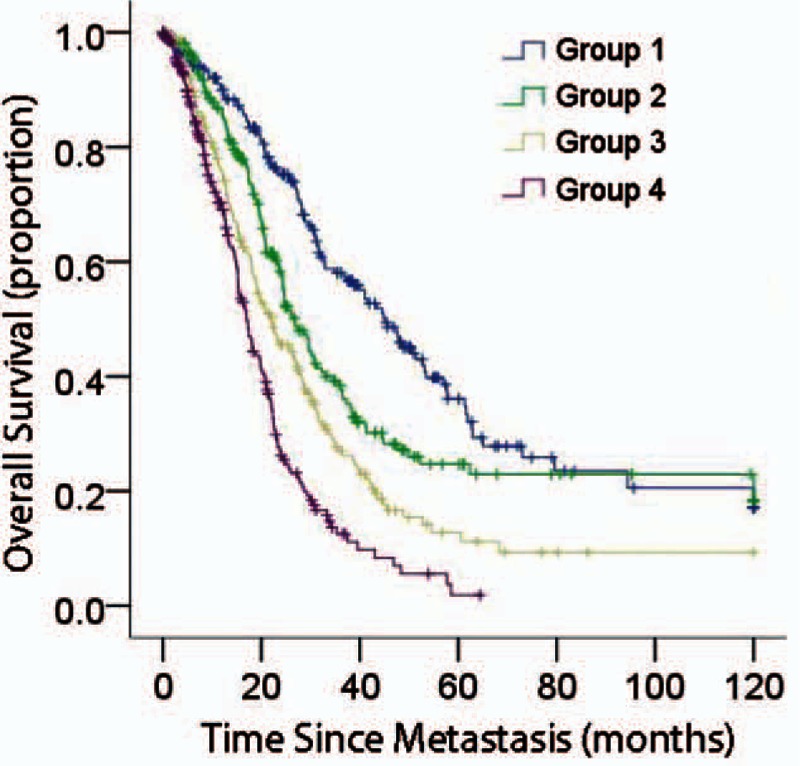
Kaplan–Meier curves for all four groups based on the linear predictor from nomogram.

In addition, we subdivided the study cohort into 4 groups based on the linear predictor of nomogram and we plotted Kaplan–Meier curves (Fig. 3). Group 1 (total points [TPs] <320, 208 patients) had the highest probability of survival as 38.3% for 3 years and 12.9% for 5 years, respectively, followed by group 2 (320 <= TP < 360, 226 patients; group 2/group 1, hazard ratio [HR] = 1.61, 95%CI: 1.24–2.09, *P* < 0.01) as 24.2% and 8.1% for 3 and 5 years, respectively, group 3 (360 <= TP < 410, 202 patients; group 3/group 1, HR = 2.20, 95%CI: 1.69–2.86, *P* < 0.01) as 16.1% and 3.7% for 3 and 5 years, respectively, and group 4 (TP >= 410, 224 patients; group 4/group 1, HR = 3.66, 95%CI: 2.82–4.75, *P* < 0.01) as 5.1% and 4.7% for 3 and 5 years, respectively. The discrimination was showed by the differences in survival between these 4 groups (Table 3).

**Table 3:**
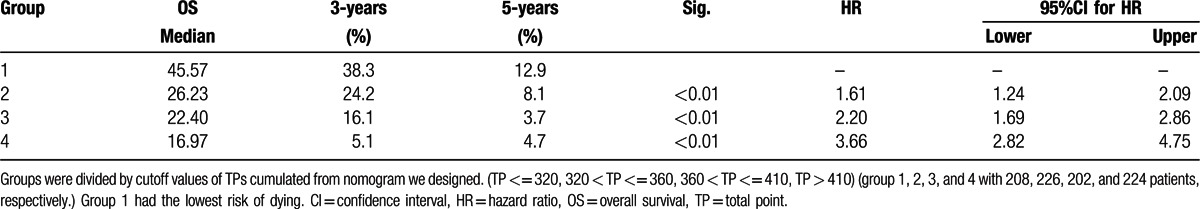
Cox regression analysis for groups based on the model.

## 4 Discussion

We developed a prognostic model predictive of 3-year and 5-year OS after subsequent metastases of patients with primary NPC. Our model was built on a metachronous metastatic cohort of 860 eligible NPC patients. In accordance with previous literatures, we found 8 independent predictors associated with metastatic OS of study cohort. Furthermore, the Cox regression models were visualized in nomogram, which might facilitate individualized prediction for future consultation.

A few prognostic models were designed for metastatic NPC patients in previous studies. Ong et al^[13]^ in 2003 built a prognostic index score (PIS) for metastatic NPC based on 6 independent negative factors: liver metastasis, lung metastasis, anaemia, poor PS, distant metastasis at initial diagnosis, and a disease-free interval (DFI) of <6 months. Khanfir et al^[14]^ in 2007 found out that poor performance status (PS), multiple metastatic sites, and prior chemotherapy were independently significant negative prognostic factors in multivariable analysis for metastatic NPC. Furthermore, Jin et al^[15]^ in 2012 developed a prognostic score model (PSM) incorporating some biomarkers, based on PS, age, hemoglobin level, LDH level, ALP level, and serum Epstein–Barr virus (EBV) DNA level for metastatic NPC in epidemic area. Kumar et al^[16]^ in 2014 evaluated scoring system and prognostic factors in patients with spinal metastases from NPC, finding out that general condition (*P* < 0.01), visceral metastases (*P* < 0.01), and vertebral metastases (*P* < 0.01) showed significant association with survival.

NPC patients with methchronous metastatic differ from that of synchronous in the heterogeneity of survival. Our previous study on patients with synchronous metastatic^[7]^ NPC revealed that the number of metastatic lesions and liver involvement are the most important prognostic factors. Screening out a subgroup of NPC patients with synchronous single metastases in nonhepatic location might benefit more from aggressive treatment. However, situations differ for patients with metachronous metastasis, for most of whom the aggressive treatment might benefit and more treatment options might be available without concerning of primary lesion, and as a result survival seemed more heterogeneous. We felt it valuable to discuss the heterogeneity of survival for the metachrous metastatic NPC patients.

Our model incorporated 8 independent predictors based on a metachronous cohort, covering general and geographic conditions of patient (age, KPS), anatomical characteristics from TNM staging (N), literature-reported biochemical markers (sLDH),^[17,18]^ and metastatic characteristics (primary metastasis to bone, primary metastasis to liver, multiple lesions, and number of locations).

Nomogram visualizes Cox regression model and facilitates individualized risk prediction in a variety of cancers.^[19–21]^ A rapid computation, precise assessment, and pellucid prognosis might facilitate better interpretation of disease and clinical decision-making.

However, there were limited studies on nomogram designed for metachronous metastatic NPC patients. Liang W et al^[22]^ in 2014 developed a nomogram to predict OS in 1520 nonmetastatic NPC patients. And Cho et al^[23]^ in 2015 developed and external validated nomogram for OS (age, performance status, smoking status, and N classification) in both synchronous and metachronous metastatic patients. Whereas on metastatic OS, we observed that metastasis related predictors – number of metastatic locations and number of lesions of locations – impact more, compared with age, UICC N stage, and KPS. Smoking status^[24–26]^ was proved of prognostic impact on OS for NPC patients, especially for male patients. Whereas, smoking was observed of weaker impact on OS, compared with other predictors such as serum LDH, age, N stage, etc. and was finally ruled out in multivariable analysis.^[27–29]^

Although based on prospective studies, nomogram might present perspective value in proposing treatment protocol. For patients with higher TP computed from nomogram intergrating all known vital factors for prognosis, aggressive treatment might worth commencing. However, for low TP patients, palliative treatment might benefit more. Thus, we believe that the utilizing of powerful predictors confirmed in previous studies and also observed in our study might help approach a little bit further for precise prediction.

Recent studies reported similar findings that EBV DNA level was a powerful prognosticator for OS, while in our study EBV DNA was ruled out based on the results of multivariate analysis.^[30–32]^ As the PCR-based techniques in EBV DNA assay vary in sensitivities, even using the same primer and sets of experimental conditions, great discrepancies in the median concentration among different studies. The cut-off varies widely in different study groups and different studies from the same study group as well.^[33,34]^ According to the time span of our study design, we failed to collect full comparable data of EBV DNA copies in our study cohort. The ongoing international effort to harmonize the assay may facilitate future exploration.

Another concern of our model could be that the amount of residual tumor after (CT) is not an independent prognostic factor, while in previous studies it is believed related to tumor biology and survival of patients.^[35]^ Nevertheless, there might be confounding factors. Aside from amount of residual tumor after CT, the location, extend, biological behavior of the residual tumor, as well as the general condition of the patients, the preferences and experiences of doctors and the feasibility of treatment might affect the final protocol and OS.^[36,37]^ Functioning as a “gray box,” for a prediction model this issue does not matter because our goal is to accurately predict survival rather than prove a causal relationship between survival and residual tumor. Predicting survival is perfectly possible when residual tumor functions as a surrogate predictor instead of an independent predictor.

There are several limitations of this study. First the nomogram was developed based on a retrospective cohort in a regionally based population in a single institute. Second, the heterogeneity in treatment protocol for metachronous metastasis patients might bring confounding effects. Finally, most patients with metastasis were diagnosed by imaging modalities, while limited patients had pathologically proofs, which could be potential source of bias. Therefore, prospective studies and external validation in multiinstitute are needed in the future.

## 5 Conclusions

We built a nomogram predictive of OS after metastasis based on the independent prognostic factors of OS from a metachronous metastatic cohort. Multiinstitutional external validation of the nomogram might be needed in the future.
